# Development and validation of rapid magnetic particle based extraction protocols

**DOI:** 10.1186/1743-422X-11-137

**Published:** 2014-08-03

**Authors:** Andrea Aebischer, Martin Beer, Bernd Hoffmann

**Affiliations:** 1 Institute of Diagnostic Virology, Friedrich-Loeffler-Institut, Suedufer 10, 17493 Greifwald-Insel Riems, Germany

**Keywords:** Nucleic acid extraction, Magnetic particle, Schmallenberg virus, Bovine viral diarrhea virus, Mobile diagnostic laboratory

## Abstract

**Background:**

In order to control and eradicate transboundary animal diseases, early diagnosis and reaction is essential for the implementation of control activities. Thus, mobile diagnostic units which allow analytical testing close to the site of occurrence could provide valuable support for centralized laboratories. Consequently, the availability of diagnostic tests using mobile amplification and detection technologies has been increasing over the past years. However, methods enabling rapid and simple nucleic acid extraction also under resource-limited settings are still scarce.

**Methods:**

In the present study rapid extraction protocols based on magnetic particle technology have been developed. For this purpose, the two open extraction platforms KingFisher™ Duo (Thermo Fisher Scientific) and BioSprint® 15 (Qiagen) as well as the fully automated EZ1® advanced XL instrument (Qiagen) were used. All protocols were validated in comparison to standard manual extraction using blood and serum samples from animals infected with Schmallenberg virus or bovine viral diarrhea virus.

**Results:**

All newly developed protocols allowed a complete extraction within 30 minutes of time. The fully automated EZ1-extraction yielded the highest reproducibility, whereas slightly higher intra- and inter-assay variations were observed using the open platforms. Compared to the manual procedure, the analytical sensitivity of all the rapid protocols was 1 log_10_ step reduced for extraction from blood samples. For sera a reduced dynamic range could only be observed using the maximally shortened BioSprint 15 protocol. Validation using clinical samples showed an excellent concordance of all the rapid extraction protocols to the standard manual extraction procedure, independent of sample materials and target viruses.

**Conclusions:**

The results of this study show that the speed-optimized novel extraction protocols allow rapid and simple nucleic acid extractions for a variety of target viruses without significant loss of sensitivity compared to standard procedures. For this reason they represent valuable tools to accelerate magnetic particle based automated extraction.

## Background

Transboundary animal diseases, as e.g. Foot-and-Mouth disease (FMD) and Classical swine fever (CSF) can have serious socio-economic consequences for affected countries. Thus, early recognition and reaction to disease outbreaks is essential to carry out control activities. In such emergency situations, the set up of local disease control centers as well as the use of mobile diagnostic units would provide valuable support for centralized laboratories. In addition, analytical testing close to the site could circumvent the delays associated with the transportation of samples and thus significantly reduce the time to achieve a test result. This would enable a more rapid local decision making which is crucial to prevent further spreading of the disease. Consequently, over the past years the availability of mobile molecular testing systems has been increasing [1-5]. The most promising developments provide fully automated sample-in/answer-out analyses by combining nucleic acid extraction, amplification and detection into one integrated system, as e.g. the GeneXpert® by Cepheid [6,7], IQuum’s Liat™ Analyzer [8,9] or the Enigma® ML [10]. Other approaches aimed to completely omit the nucleic acid extraction step and focused on the development of robust PCR systems that are less prone to inhibition, allowing amplification of target genes directly from crude samples. A variety of direct PCR assays have already been described and successfully applied [11-13]. However, the vast majority of the available amplification and detection technologies require off-line sample preparation and purification of the target nucleic acids. [1,4,14]. For this purpose, a broad spectrum of well validated manual and automated extraction technologies is already available. However, many of these methods involve lengthy procedures that often have to be performed by a trained technician. Alternatively, sophisticated automated extraction robots allow high-throughput analyses and significant reduction of hands-on time per sample. Opposed to that, rapid and simple extraction protocols that can also be performed under resource-limited settings, as e.g. directly in the field, are still scarce.

In the present study we aimed to establish rapid protocols which can be operated on various extractions instruments and which can be easily adapted to a broad spectrum of downstream assays. The protocols were developed using the two open extraction platforms KingFisher™ Duo (Thermo Fisher Scientific Inc.) and BioSprint® 15 (Qiagen) as well as the fully automated EZ1® Advanced XL instrument (Qiagen). Optimization was performed for each platform separately in order to reach the shortest possible processing times while maintaining reliable and reproducible pathogen detection.

For the initial validation of the protocols two RNA viruses have been selected. Schmallenberg virus (SBV) is an *Orthobunyavirus* from the family *Bunyaviridae* and belongs to the Simbu serogroup [15,16]. Monitoring of infected animals is performed in many countries inside and outside of the European Union in order to increase the knowledge on epidemiology and pathogenesis of this newly emerging disease [17,18]. Bovine viral diarrhea virus (BVDV) is classified as a member of the genus *Pestivirus* within the family *Flaviviridae*[19]. It has been shown before, that detection of persistently infected cattle using rapid and simple test systems is a valuable tool in BVD-eradication programs [20,21].

The performance of the speed-optimized extraction protocols was further investigated with regard to the efficiency of RNA recovery, reproducibility, as well as analytical and diagnostic sensitivity. In addition, characteristics like complexity, hands-on and total processing time were evaluated in order to assess the suitability of the methods for application under resource-limited settings.

## Materials and methods

### Samples

Negative samples as well as all the SBV-positive blood and serum samples were obtained during animal trials at the Friedrich-Loeffler-Institut (FLI, Germany). All experimental protocols were reviewed by a state ethics commission and have been approved by the competent authority (State Office for Agriculture, Food Safety and Fisheries of Mecklenburg-Vorpommern, Rostock, Germany, ref. LALLF M-V TSD/7221.3-1.1-004/12). BVDV-positive samples were kindly supplied by veterinary laboratories of the German federal states. All samples were aliquoted and stored at -20°C until further processing. Additional samples positive for classical swine fever virus (CSFV) and bluetongue virus (BTV) were provided by the respective national reference laboratories at the FLI.

### Manual extraction

Manual extraction was performed using the QIAamp Viral RNA Mini Kit (Qiagen, Hilden, Germany) according to the manufacturer’s instructions. The input volume was 100 μl for serum and 75 μl for blood samples. Extracted RNA was eluted in 100 μl of Buffer AVE.

### Automated rapid extraction

#### Strategy

Rapid protocols were established on three magnetic particle based extraction instruments. This included (i) the selection of suitable extraction reagents for all systems and (ii) the design of maximally shortened extraction protocols for the open extraction platforms.

A variety of extraction reagents commercially available from Qiagen, Macherey-Nagel (Düren, Germany) and LSI/life technologies (Lissieu, France) were comparatively evaluated in order to select the most suitable chemistry. For this purpose, an established protocol for magnetic particle based extraction was used (Original protocol, Table 1).

**Table 1:** Speed-optimized extraction protocols for the KF Duo and the BS 15 instrument

**Step**	**Reagents**	**Timing of protocol steps (min)**		
		**Original**	**KF Duo**	**BS 15**
Binding	VXL + ACB + Beads	5	2	2
Wash 1	AW1	3	12 s	1
Wash 2	AW2	2	12 s	1
Wash 3	Ethanol 100%	2	12 s	-
Evaporation	Water	5	0	0
Elution	AVE	2	1	0.5
Total time		24:00	8:00	7:50

Each step of the original protocol was shortened to the maximum for the respective extraction platform.

The BindIt Software v3.2 (Thermo Fisher) was used to design protocols with the shortest possible processing time for the two open extraction platform. For this purpose, each step of the original protocol indicated in Table 1, was shortened progressively to the maximum. Evaluations were performed using SBV-positive blood samples and successive RT-qPCR detection of virus-specific and heterologous internal control RNA.

#### Extraction platforms

##### EZ1® Advanced XL (Qiagen, Hilden, Germany)

The EZ1 Advanced XL is a closed extraction system for up to 14 samples using pre-filled reagent cartridges and pre-programmed protocols. For extraction, the EZ1 DNA Blood 200 μl kit (Qiagen) was used, but the reagents of the supplied cartridges were exchanged with the following buffers: binding buffer ACB, washing buffers AW1 and AW2, and elution buffer AVE (Qiagen) [see Additional file 1: Table S1]. For lysis, 200 μl of the sample material were mixed with 200 μl of lysis buffer VXL (Qiagen) and the lysate was loaded to the EZ1 instrument. Automated extraction was then performed immediately using the EZ1 Advanced DNA Blood Card (Qiagen) which has a processing time of 16 min. The extracted RNA was eluted in 100 μl.

##### KingFisher™ Duo system (Thermo Fisher Scientific Inc., Waltham, MA, USA)

The KingFisher™ (KF) Duo system was applied with a 12 pin magnet head which enabled processing of 12 samples per run using microtiter 96 deepwell plates (Thermo Fisher). Prior to the extraction process, the deepwell plates were filled with the following reagents: lysis buffer VXL, MagAttract Suspension B, buffer AW1, buffer AW2 (Qiagen), 100% Ethanol (Carl Roth GmbH, Karlsruhe, Germany), nuclease-free water and buffer AVE (Qiagen) [see Additional file 1: Table S1]. For extraction, 100 μl of the sample were added to the lysis buffer and mixed by pipetting. After that, binding buffer ACB was added and automated extraction was executed and completed within 8 min using the protocol indicated in Table 1. The extracted RNA was eluted in 100 μl and subsequently transferred to 1.5 ml tubes for storage.

##### BioSprint® 15 workstation (Qiagen)

The BioSprint 15 (BS 15) workstation is an open extraction platform for processing of up to 15 samples per run using 5-tube plastic strips (Qiagen). The system is identical to the KingFisher™ mL which is produced by Thermo Fisher Scientific, Inc. The extraction was performed as described before for the KF Duo using the respective protocol described in Table 1. For details on pre-filling of 5-tube strips see Additional file 1: Table S1. The total running time to complete the BS 15 extraction was 7.5 min.

### RT-qPCR

For RT-qPCR detection of SBV-RNA, a previously published assay was used [22]. For the BVDV assay primers Pesti-3F and Pesti-3R [23] were used in combination with the TQ-Pesti probe [24]. Detection of heterologous internal control RNA was conducted as described before [25]. All reactions were performed in a 12.5 μl volume using the AgPath-ID™ One-Step RT-PCR kit (Applied Biosystems, Carlsbad, CA, USA) according to the manufacturer’s instructions. RT-qPCR was performed on a Bio-Rad CFX96 Real-Time Detection System (software Bio-Rad CFX Manager 3.0; Bio-Rad, Hercules, CA, USA) or on an Eco™ Real-Time PCR System (Eco™ software v4.0; amplifa Labortechnik GmbH, Wasserburg, Germany) using the following thermal profile: reverse transcription for 10 min at 45°C, activation of polymerase for 10 min at 95°C, followed by 45 cycles of denaturation for 15 s at 95°C, annealing for 20s at 56°C and elongation for 30s at 72°C.

### Study design

**Figure 1:**
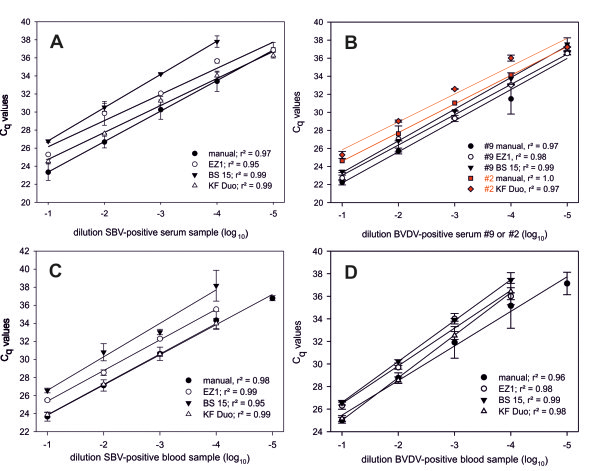
**Analytical sensitivity and linear dynamic range of manual and rapid automated extraction methods.** Dilution series of serum samples positive for SBV-RNA **(A)** or BVDV-RNA **(B)** as well as dilution series of EDTA-blood samples positive for SBV-RNA **(C)** or BVDV-RNA **(D)** were used to evaluate the extraction efficiency of the rapid extraction protocols in comparison to the standard manual extraction protocol. Each dilution step was extracted in duplicate and the extracted RNA from each panel was tested in the same RT-qPCR run. R^2^ values as defined by regression analysis are indicated for each method.

(1) The relative sensitivity and the dynamic range of the rapid extraction protocols were determined using 10-fold dilution series of SBV- or BVDV-positive blood and serum samples. Dilutions were prepared in blood or serum tested negative for the respective pathogen, aliquoted and stored at -20°C until processing. For each extraction technique duplicates of the different dilutions were extracted in parallel. Due to the small volume of the provided serum samples, two different BVDV-positive samples had to be used (number 2 and number 9, as indicated in Figure 1B).

(2) To assess the intra- and inter-run variation of each extraction method, one SBV-RNA-positive EDTA blood sample was extracted 6 times in each of three independent runs.

(3) A panel of clinical samples was used to assess the performance of the rapid extraction procedures in comparison to the standard manual extraction protocol. The panel consisted of 26 SBV-positive (14× EDTA blood, and 12× serum) and 24 BVDV-positive samples (12× EDTA blood, 12× serum). Each sample was extracted once using the QIAamp Virus Mini Kit and in duplicate with each of the rapid automated protocols.

(4) BTV- and CSFV-positive samples were analyzed using the BS 15 and the EZ1 to investigate the suitability of the procedures for extraction of additional target viruses.

### Statistical analysis

Regression analysis was used to assess the dynamic range and the linearity of each extraction method. Pearson’s correlation coefficient was calculated to compare the analytical performance of both the automated and the manual extraction. Differences in mean C_q_-values were analyzed by the parametric or non-parametric paired *t*-test in order to assess differences in the extraction efficiency concerning sample material and target virus. P-values ≤ 0.05 were considered as significant. All analyzes were performed using the SigmaPlot software v11. PCR efficiencies were calculated from the slopes of standard curves indicated in Figure 1 using the equation E = 10^(-1/sope)^ – 1.

## Results

### Selection of extraction reagents

In order to select an optimal chemistry, lysis buffers from different commercially available extraction kits were comparatively evaluated. Emphasis was set on the potential of the buffers to provide sample lysis at room temperature without additional incubation time. Lysis buffer VXL (Qiagen) outperformed the remaining candidates with regard to these properties and was therefore selected as a basis for the rapid extraction protocols. Optimal lysis was obtained by using a 1:1 mixing ratio of sample and buffer VXL. For serum and blood samples volumes of 100 μl (KF Duo and BS 15) or 200 μl (EZ1) proved to be optimal.

### Design of rapid extraction protocols

Since the EZ1 is a closed extraction system, modification of extraction protocols by the user is not possible. Therefore, we selected the EZ1 Advanced DNA Blood Card (Qiagen) for our experiments which provided the shortest processing time among the commercially available protocols.

A standard protocol for magnetic particle based extraction was used as basis for the KF Duo and BS 15 procedures. For both extraction platforms, the initial binding step could be reduced from 5 min to 2 min. Furthermore, the original ethanol evaporation step was replaced by a water rinse prior to elution of the extracted RNA. Due to the different layouts of the extraction platforms (96 well plates or 5-tube strips, respectively) the washing times had to be optimized separately for the KF Duo and the BS 15. For each platform the shortest possible times which still guaranteed a reliable and reproducible extraction have been determined. Whereas the three washing steps of the KF Duo could be reduced to 12 s each, the two washing cycles of the BS 15 required processing times of 1 min in order to guarantee reliable results (Table 1). Elution time was found to be critical for reproducibility of the extraction performance and was also optimized separately for each platform (Table 1).

### Reproducibility

Reproducibility of the rapid extraction protocols and the standard manual extraction was assessed using 6 replicates of one SBV-positive blood sample in 3 independent runs. Mean C_q_-values as well as intra- and inter-assay coefficients of variation are shown in Table 2. The highest reproducibility was found for the automated EZ1-extractions, whereas the highest intra- and inter-assay variations were observed for the BS 15.

**Table 2:** Reproducibility of manual and rapid automated extraction techniques

**Extraction**	**Mean C**_**q**_**value**	**Intra-run**		**Inter-run**	
		**SD**	**CV%**	**SD**	**CV%**
Manual	23.83	0.19	0.80	0.14	0.58
EZ1	24.10	0.16	0.65	0.12	0.51
BS 15	25.12	0.34	1.36	0.28	1.10
KF Duo	25.31	0.25	1.00	0.20	0.79

SD = standard deviation, CV = coefficient of variation.

Mean C_q_ values, standard deviations of replicates as well as intra- and inter-assay coefficients of variation were defined using 6 replicates of an SBV-positive blood sample in 3 independent runs.

### Linearity and analytical sensitivity

The dynamic range of each extraction method was investigated using 10-fold dilution series of BVDV- or SBV-positive blood and serum samples. For serum samples, target RNA was detected over a concentration range of 5 log_10_ steps by manual, EZ1- and KF Duo-extraction. Using the BS 15 the linear dynamic range covered only 4 log_10_ steps in case of SBV (Figure 1A, 1B). For blood samples, the analytical sensitivity of all the rapid extraction procedures was 1 log_10_ step reduced compared to the manual extraction protocol (Figure 1C, 1D). Standard curves and regression analysis revealed a linear response for all the investigated extraction techniques and excellent quantitative parameters (Figure 1). Based on the slopes of standard curves, the PCR efficiencies were calculated for the respective RNA extracts (Table 3). The EZ1- and KF Duo-extracts yielded similar or even higher PCR efficiencies than the concordant manually extracted samples. A reduction in PCR efficiency was observed for all BS 15-extractions, independent of sample material and target virus, as well as for the extraction of BVDV-RNA from blood using the KF Duo (Table 3).

**Table 3:** PCR efficiency after manual and automated extraction

**Target**	**Material**	**% PCR efficiency**			
		**Manual**	**EZ1**	**BS 15**	**KF Duo**
SBV	Blood	99.6	97.0	86.1	101.2
	Serum	97.8	121.5	87.1	112.7
BVDV	Blood	112.4	99.3	88.8	84.2
	Serum	95.0	98.2	92.8	110.8

10-fold dilution series of SBV- and BVDV-positive blood and serum samples which were extracted in duplicate; PCR efficiencies of the respective RNA extracts were calculated from the slope of the standard curves.

### Clinical samples and correlation with manual extraction

**Figure 2:**
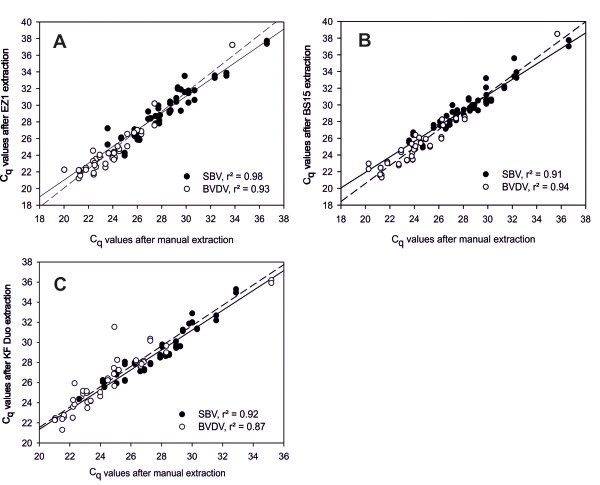
**Analytical performance of rapid automated extraction using clinical samples.** Direct comparison of standard manual extraction with automated extraction procedures using the EZ1 Advanced XL **(A)**, the BS 15 **(B)**, and the KF Duo **(C)**. For this purpose, blood and serum samples positive for SBV-RNA (black circles) or BVDV-RNA (white circles) were used. Regression is indicated by solid lines for SBV-positive samples and by dashed lines for BVDV-positive samples.

The performance of the rapid extraction procedures in comparison to manual extraction was further evaluated using blood and serum samples from animals experimentally infected with SBV or naturally infected with BVDV. EZ1-extraction (Figure 2A) of SBV-RNA showed a very good correlation to the manual procedure (r = 0.95) with an average difference of C_q_-values (∆C_q_) of -1.08 (standard deviation SD 0.98). For extraction of BVDV-RNA the ∆C_q_ was -0.65 (SD 0.98) with a total correlation coefficient of 0.96*.* Extractions on the BS 15 (Figure 2B) yielded a very good correlation to manual extraction for SBV (r = 0.96) and BVDV (r = 0.97) with corresponding ∆C_q_ values of -1.31 (SD 0.83) and -0.92 (SD 0.86), respectively. Using the KF Duo protocol (Figure 2C), the total correlation to manually extracted samples was slightly better for SBV-RNA (r = 0.96) compared to BVDV-RNA (r = 0.94) which corresponds to ∆C_q_ values of -1.25 (SD 0.71) for SBV and -1.6 (SD 1.11) for BVDV.

For all procedures, extraction of target RNA was equally efficient from blood and serum samples.

Using the BS 15 and the EZ1, BVDV-RNA was recovered with a significantly higher correlation to the manual extraction than SBV-RNA (P < 0.05). For all samples extracted on the KF Duo heterologous internal control (IC) RNA was added to the lysates and extracted in order to gain additional insights into the quality of the purification process. After manual extraction, IC-RNA was detected in blood and serum samples with a mean C_q_-value of 27.7 (SD 1.09) or 26.6 (SD 0.36), respectively, Using the KF Duo, the mean C_q_-value was 29.6 (SD 1.28) for blood samples and 27.9 (SD 0.6) in case of serum samples.

In order to investigate the suitability of the EZ1- and the BS 15-protocol for extraction of different target viruses, BTV- and CSFV-positive samples were also analyzed. Using BTV-positive blood samples, a very good correlation to manual extraction could be found for both, the EZ1 (r = 0.95) and the BS 15 (r = 0.98) [see Additional file 2: Figure S1]. Similar results were obtained for CSFV-positive serum samples. Even though the analytical sensitivity was 1 log_10_ step reduced using the rapid protocols, r-values of 1.00 for the EZ1 and 0.98 for the BS 15 again indicated an excellent concordance with the manual extraction protocol [see Additional file 3: Figure S2].

### Operational analysis: hands-on time and total processing time

The hands-on and total processing time of each extraction method was measured using a set of 8 samples (Table 4). The total hands-on time required to perform each step of the extraction procedure was about 16 min for the KF Duo and the BS 15, which included the time used for filling deepwell plates or tube strips. Thus, together with an instrument running time of about 8 min, one extraction run could be completed within 25 min. In order to reduce the total processing time to 20 min, pre-filled and sealed deepwell plates or tube strips could be used. Using the EZ1, 41 min were required for the extraction of 8 samples. However, the main part of the 25 min hands-on time was needed to empty and re-fill the reagent cartridges. By using pre-filled cartridges, which contain all necessary reagents, the extraction process could be reduced to less than 30 minutes.

**Table 4:** Operational analysis of manual and automated extraction procedures

**Operator step**	**Required operator time (minutes)**			
	**Manual**	**EZ1**	**BS 15**	**KF Duo**
Filling of reagents	5^*a*^	15	5	5
Mix sample + lysis buffer	10	5	10	10
Incubation	10	0	0	0
Load instrument	0	5	1	1
Extraction/Instrument run	30	16	7.5	8
Total hands-on time	40 - 45	25	16	16
Total processing time	50 - 55	41	24	24

^*a*^dissolving carrier RNA.

The time required for each operator step was measured for the extraction of 8 samples, respectively.

## Discussion

Over the past years, the demands for rapid and simple diagnostic tests which can be performed outside of a centralized laboratory have been increasing. However, despite the increasing availability of innovative mobile amplification and detection technologies, the sample processing step remains an important bottleneck in these developments. In the present study we established rapid extraction protocols which can be operated on different portable extraction instruments. The protocols were optimized in order to reach the shortest possible processing times which still allowed reproducible and reliable pathogen detection.

Among a variety of extraction reagents from different suppliers, we selected the lysis buffer VXL (Qiagen) as a basis for our extraction protocols because it promoted efficient cell lysis without any incubation time simply by mixing it thoroughly with the sample. Subsequent addition of binding buffer ACB (Qiagen) to the lysate further enabled a very rapid binding of the extracted nucleic acids to the magnetic particles. Since it does not require any additional equipment, as e.g. a heating block, the method is suitable for application under resource-limited settings in emergency situations. It further represents an attractive tool to decrease the processing time of magnetic particle based extractions also in the laboratory routine. The KF Duo protocol can be applied without further modification on similar platforms with higher capacity, as e.g. the KingFisher™ Flex system (Thermo Fisher). Thus, extraction of up to 96 samples could be completed in less than 30 min of time.

The intra- and inter-assay variations of the rapid extraction protocols were found to be in the same range as those of a standard extraction method. This indicates that a reliable and reproducible nucleic acid extraction is possible despite a marked shortening of the whole purification procedure. Due to the fully automated and standardized working process, the lowest variability was found for the EZ1-extraction. Slightly higher intra- and inter-assay variations were observed for the two open extraction platforms, most likely since these systems required more user input and manual pipetting steps. It was further seen, that the KF Duo performed better than the BS 15, which might be associated with the additional Ethanol-washing step included in the KF Duo protocol made possible by the use of 96 well deepwell plates for extraction.

Using dilution series of RNA-positive samples, the rapid extraction protocols yielded a high accuracy and linearity compared to the “gold standard” manual procedure. However, differences could be observed between the individual systems. Extraction on the BS 15 caused a reduction in PCR efficiency independent of the applied sample material and thus showed the lowest analytical sensitivity among the three evaluated methods. This can be associated with the shortening of the washing process to only 2 steps opposed to the 3 steps included in the EZ1- and the KF Duo-protocol. However, the latter two protocols revealed variations in extraction efficiency dependent on the specimen used for extraction. From serum samples, RNA could be recovered over the same dynamic range as with the standard manual extraction protocol. In contrast, the analytical sensitivity was one log_10_ step reduced for blood, a fact which could be explained with a high content of PCR inhibitory substances [26,27]. Most likely, the inhibitors cannot be completely removed from the final RNA extracts using the shorter purification procedures. Thus, residues of these substances could interfere with the subsequent PCR amplification step of the target genome resulting in a negative influence on the test sensitivity. Accordingly, monitoring of internal control RNA during KF Duo extraction showed an inferior quality of the RNA extraction compared to the standard manual procedure. In order to investigate the relevance of these findings for a future field-use, clinical samples from naturally as well as experimentally infected animals were analyzed. Independent of the sample material and its quality, the three rapid extraction techniques yielded an excellent correlation with the selected standard manual procedure. Hence, the slightly inferior analytical sensitivity and extraction quality described above are not critical with regard to a point-of-care application since clinically diseased animals with high genome copy numbers of the respective pathogens are the main targets.

Using the EZ1 and the BS 15, RNA extraction was more efficient for BVDV than for SBV, independent of the applied sample material. This finding might indicate that the non-segmented BVDV-genome is easier to recover than the tripartite SBV-genome using the short extraction procedures. The suitability of the rapid protocols for extraction of different target viruses was therefore investigated in more detail by testing additional BTV- and CSFV-positive samples. In accordance to the results obtained for SBV and BVDV, an excellent correlation to the manual extraction procedure could be demonstrated for both, the EZ1- and the BS 15-protocol. This implies that the newly developed protocols can be applied for a broad range of downstream assays without further adaptations dependent on the target virus. Thus, simultaneous screening for different pathogens can be performed after a single preparation step. However, with regard to practicability of these techniques, there are several important factors to consider besides their analytical performance. This includes complexity of operation, hands-on time, total processing time as well as necessary technical skills of the operator.

Using the EZ1 demonstrated that a closed extraction system could result in a markedly reduced hands-on time. Except for the initial mixing of the sample with the lysis buffer, all pipetting steps are automatically performed by the instrument, which allows a high standardization. However, to enable extraction of viral RNA using the EZ1 Advanced DNA Blood Card we had to use the card in combination with modified reagent cartridges that are not commercially available. Thus, a successful application of the EZ1 in a rapid extraction protocol is dependent on the supply of suitable extraction kits by the manufacturer. In our view, this lack of flexibility represents a significant drawback of the closed system. Since the instrument can only be operated with pre-programmed cards and accompanying kits, its use is limited to specified applications and associated with relatively high costs per reaction.

Contrary, flexibility is the most attractive feature of the open extraction platforms, i.e. the protocols can easily be modified and adapted to a broad range of applications and pathogens. Furthermore, chemistries can be freely selected and combined which allows a more specific optimization and a reduction of costs per reaction. Even though we used buffers VXL and ACB as a basis, the presented protocols could also be operated and validated using similar extraction reagents from other suppliers. In comparison to the EZ1, the KF Duo and the BS 15 required more direct user-input. This included several operator steps like pre-filling of deepwell plates or tube-strips, preparation of lysates, addition of binding buffer, or transfer of extracted RNA to storage tubes. Hence, a certain degree of technical skills is required for the operation and the risk of human errors and contaminations is comparatively higher than with a closed system. However, an additional advantage of the KF Duo and the BS 15 is the relatively small size and low weight of the instruments (16 kg and 10 kg, respectively). For the EZ1, which has a weight of about 50 kg, transportation and operation in a car might be more challenging.

## Conclusion

In the present study, three rapid automated extraction protocols were developed and validated. Using two open platforms provided the possibility to freely adapt chemistry and to design protocols with a minimum processing time. In contrast, the fully automated EZ1 system required a minimum of hands-on time, showed a high reliability and a reduced risk of contamination. The newly developed protocols allowed nucleic acid extraction in less than 30 min and showed excellent concordance with a standard manual extraction protocol. For this reason, they represent valuable tools for rapid sample processing under emergency settings as well as for the acceleration of magnetic particle based extractions in the routine laboratory diagnostic.

## Competing interests

The authors declare no conflict of interest. Qiagen-Germany was a collaboration partner in the presented research project.

## Authors’ contributions

AA and BH designed the study; AA made the experiments, AA, BH and MB wrote the paper. All authors read and approved the final manuscript.

## Supplementary Material

Additional file 1: Table S1 Reagents and filling scheme for rapid automated extraction protocols. Detailed list of the reagents and the volumes used to prefill deepwell plates or 5-tube strips for extraction on the KF Duo and the BS 15, as well as reagents used to create the proprietary reagent cartridges for EZ1 extraction. Click here for file

Additional file 2: Figure S1 Evaluation of EZ1- and BS 15-extraction using BTV-positive samples. 8 BTV-positive blood samples covering serotypes 2, 4 and 8 were extracted with each extraction technique and directly compared to the standard manual extraction protocol. The corresponding regression lines are indicated solid for the EZ1 and dashed for the BS15. Click here for file

Additional file 3: Figure S2 Evaluation of EZ1- and BS 15-extraction using CSFV-positive samples. A dilution series of a CSFV-positive serum sample was extracted in triplicate with each extraction technique and in comparison with the standard manual procedure. Results of linear regression analysis are indicated for each method. Click here for file
