# Supplementary material for: Development and validation of rapid magnetic particle based extraction protocols
Source: Virol J. 2014 Aug 3;11:137. doi: 10.1186/1743-422X-11-137 (PMC4124660; doi:10.1186/1743-422X-11-137)
Supplement: Additional file 1: Table S1 — Reagents and filling scheme for rapid automated extraction protocols. Detailed list of the reagents and the volumes used to prefill deepwell plates or 5-tube strips for extraction on the KF Duo and the BS 15, as well as reagents used to create the proprietary reagent cartridges for EZ1 extraction. [file 1743-422X-11-137-S1.docx]

**Additional Table 1: Reagents and filling scheme for rapid automated extraction protocols**

Detailed list of the reagents and the volumes used to prefill deepwell plates or 5-tube strips for extraction on the KF Duo and the BS 15, as well as reagents used to create the proprietary reagent cartridges for EZ1 extraction.

| Platform (plasticware) | Row or well | Reagents | Volume (µl) |
| --- | --- | --- | --- |
| KingFisher™ Duo  (96 well microtiter deepwell plates) | 1 | Lysis buffer VXL  MagAttract Suspension B  *Buffer ACB ^b^* | 100  20  250 |
|  | 2 | Buffer AW1 | 500 |
|  | 3 | Buffer AW2 | 500 |
|  | 4 | Ethanol 100% | 500 |
|  | 5 | Water | 500 |
|  | 6 | Buffer AVE | 100 |
|  | 7 | Empty |  |
|  | 8 | Empty |  |
| BioSprint® 15  (5-tube strips) | 1 | Lysis buffer VXL  MagAttract Suspension B  *Buffer ACB ^b^* | 100  20  250 |
|  | 2 | Buffer AW1 | 700 |
|  | 3 | Buffer AW2 | 700 |
|  | 4 | Water | 500 |
|  | 5 | Buffer AVE | 100 |
| EZ1® advanced XL  (Reagent cartridges of the EZ1 DNA Blood 200 µl kit) *^a^* | 1 | Buffer ACB | 500 |
|  | (2) | (MagAttract Suspension B) |  |
|  | (3) | (Bead Buffer) |  |
|  | 4 | Buffer AW1 | 900 |
|  | 5 | Buffer AW2 | 900 |
|  | 6 | Buffer AW2 | 900 |
|  | (7) | (Water) |  |
|  | 8 | Buffer AVE | 220 |
|  | 9 | Empty |  |
|  | 10 | Empty |  |

*^a^* Exchange of the pre-filled reagents, except for wells in brackets

*^b^* Binding Buffer ACB was added after mixing of lysis buffer, magnetic particles and sample
